# A Bio‐Responsive Hydrogel with Spatially Heterogeneous Structure for Treating Infectious Tissue Injuries

**DOI:** 10.1002/advs.202500088

**Published:** 2025-04-28

**Authors:** Zongtai Li, Tao Yang, Xiaolei Li, Panchao Yin, Bo Yang, Dongying Li, Yan Wang, Wei Teng, Qianqian Yu, Weichang Li

**Affiliations:** ^1^ Hospital of Stomatology Guangdong Provincial Key Laboratory of Stomatology Guanghua School of Stomatology Sun Yat‐sen University Guangzhou 510055 P. R. China; ^2^ South China Advanced Institute for Soft Matter Science and Technology School of Emergent Soft Matter Guangdong Provincial Key Laboratory of Functional and Intelligent Hybrid Materials and Devices South China University of Technology Guangzhou 510640 P. R. China

**Keywords:** bio‐responsive, hydrogel, infectious tissue injuries, polyoxometalates, spatially heterogeneous

## Abstract

Infectious tissue injuries, exacerbated by bacterial infections and antibiotic resistance, pose significant challenges for treatment and may lead to life‐threatening systemic infections. In this study, a bio‐responsive hydrogel system is developed, leveraging silver ions (Ag⁺) encapsulated in Preyssler‐type polyoxometalates (POMs). The Ag⁺ ions are selectively released in response to endogenous sodium ions (Na⁺) within the biological environment, enabling broad‐spectrum antibacterial activity. The POM serves as a protective matrix for Ag⁺, preserving its bioactivity while mitigating cytotoxicity and the reduction in antimicrobial efficacy associated with prolonged exposure. Additionally, a dual‐channel technique is employed to fabricate fiber membranes with controllable and continuously stacked chemical compositions, ensuring efficient and uniform POM incorporation via hydrogen bonding within the fiber matrix. Subsequently, in situ hierarchical cross‐linking process generated a spatially heterogeneous hydrogel with an interpenetrating network structure at multiple scales. This differentiated microstructure facilitates the controlled loading and release of diverse therapeutic components. Meanwhile, bioactive exosomes are integrated into the hydrogel, further enhancing its regenerative potential for treating infectious tissue injuries. In vitro and in vivo experiments demonstrated that the advanced hydrogel system provide a viable and efficient platform for addressing the challenges associated with infectious tissue injuries, offering a promising strategy for clinical applications.

## Graphical abstract

The hydrogel incorporates Ag⁺‐loaded Preyssler‐type polyoxometalates (POMs) that release Ag⁺ ions in response to endogenous Na⁺ ions, ensuring controlled antibacterial activity. The spatially heterogeneous structure, created through dual‐channel fiber stacking and in situ cross‐linking, enhances structural integrity and promotes tissue regeneration.

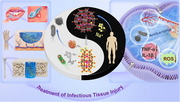

## 1 Introduction

Treating infectious tissue damage is a significant medical challenge due to bacterial infection, which delays repair, exacerbates damage, and can lead to tissue necrosis. Severe infections may escalate to systemic complications, posing a threat to life.^[^ ^1^, ^2^ ^]^ Antibiotics, although widely used, are increasingly ineffective due to the rapid emergence of drug‐resistant pathogens.^[^ ^3^ ^]^ Consequently, treating infectious tissue damage has become a prolonged and arduous process, imposing considerable economic and societal burdens.^[^ ^4^, ^5^ ^]^ Despite encouraging advancements in infection management, achieving a balance between antibacterial efficacy, infection control, immunomodulation, and tissue regeneration remains difficult.^[^ ^6^, ^7^, ^8^ ^]^ Developing efficient treatment strategies for infectious tissue damage thus remains a critical challenge in biomedicine.

Biomaterials such as membranes, sponges, scaffolds, fibers, and hydrogels are commonly utilized platforms for tissue repair.^[^ ^9^, ^10^ ^]^ Hydrogels, in particular, are attractive due to their 3D polymer networks, which mimic the extracellular matrix and provide a supportive niche for tissue regeneration.^[^ ^11^, ^12^ ^]^ Hydrogels offer tunable properties, enabling the incorporation of intrinsic biological functionalities to meet diverse therapeutic needs.^[^ ^11^, ^13^ ^]^ However, conventional hydrogels often face structural integrity issues when their polymeric networks are disrupted, compromising their functionality.^[^ ^13^ ^]^ Moreover, their hydrophilic matrices exhibit low encapsulation efficiency for hydrophobic drugs and uncontrolled release kinetics, reducing therapeutic efficacy.^[^ ^14^, ^15^ ^]^ To address these limitations, novel hydrogel structures need to be developed to meet evolving biomedical demands.

Hydrogels primarily act as coverings, supports, and barriers in treating infectious tissue damage. However, synergistic effects with functional drugs or bioactive components are essential to address complex therapeutic needs. Traditional approaches often involve antibiotics or materials loaded with antibiotics,^[^ ^16^ ^]^ but the overuse of antibiotics has led to multi‐drug resistance and side effects, limiting their effectiveness.^[^ ^16^, ^17^ ^]^ Silver (Ag), with broad‐spectrum antibacterial activity, has emerged as an effective alternative.^[^ ^18^ ^]^ However, silver's cytotoxicity at high concentrations and its reduced antibacterial efficacy due to aggregation in physiological environments remain significant challenges.^[^ ^19^, ^20^, ^21^ ^]^ Effective protection and precise release control of Ag are therefore critical to its therapeutic potential. Polyoxometalates (POMs), discrete and structurally defined oxide clusters, offer a promising solution.^[^ ^17^, ^22^, ^23^ ^]^ POMs exhibit diverse morphologies and functional properties, including nanoscale cavities and channels conducive to ion conduction, exchange, and separation.^[^ ^24^ ^]^ Their surface hydroxyl and oxo ligands, coupled with high charge density, provide opportunities for interaction with biomolecules and materials.^[^ ^25^, ^26^ ^]^ These attributes enable POMs to synergize with biomaterials for therapeutic applications.^[^ ^27^ ^]^

**Figure 1:**
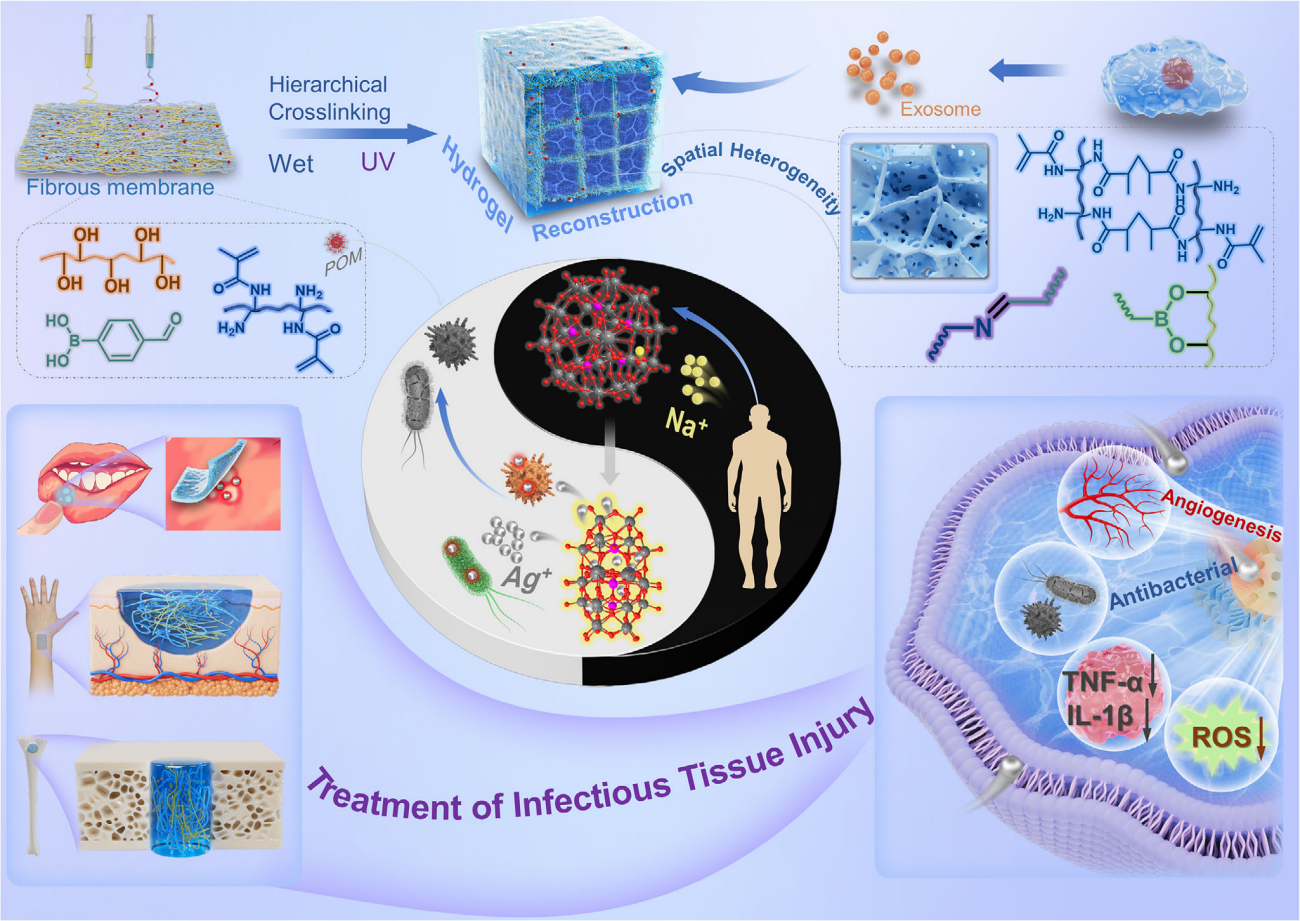
Schematic illustration of the bio‐responsive hydrogel system designed.

In this study, silver ions (Ag⁺) were encapsulated in Preyssler‐type phosphotungstates ([MP₅W₃₀O₁₁₀]^(15−n)−^) to form nanoscale AgP₅W₃₀ clusters. These clusters exhibit bio‐responsive behavior, selectively releasing Ag⁺ ions in response to endogenous sodium ions (Na⁺) within the biological environment. This Na⁺‐induced release mechanism ensures a controlled and effective antibacterial action. To stabilize the AgP₅W₃₀ clusters, polyvinyl alcohol (PVA), a hydroxyl‐rich polymer, was employed, leveraging hydrogen bonding and electrostatic interactions to prevent aggregation and preserve antibacterial efficacy. To address the clinical challenges of treating infectious tissue injuries, a spatially heterogeneous hydrogel system was developed (**Figure** **1**). This system was constructed by stacking pre‐formed PVA and methacrylated gelatin (GelMa) fibers, followed by in situ crosslinking to create a distinctive 3D network. The spatially heterogeneous architecture facilitates uniform drug dispersion, enhances structural integrity, and overcomes the limitations of conventional hydrogel systems prone to network collapse. AgP₅W₃₀ clusters were stably integrated into the PVA fibers, ensuring sustained antibacterial functionality while incorporating bioactive exosomes to promote tissue regeneration. Comprehensive in vitro and in vivo evaluations revealed the hydrogel's bio‐responsive multifunctionality, demonstrating its ability to combat bacterial infections and promote the repair of both soft and hard tissue injuries. This innovative approach provides a promising and efficient therapeutic platform for the treatment of infectious tissue damage.

## 2 Results and Discussion

**Figure 2:**
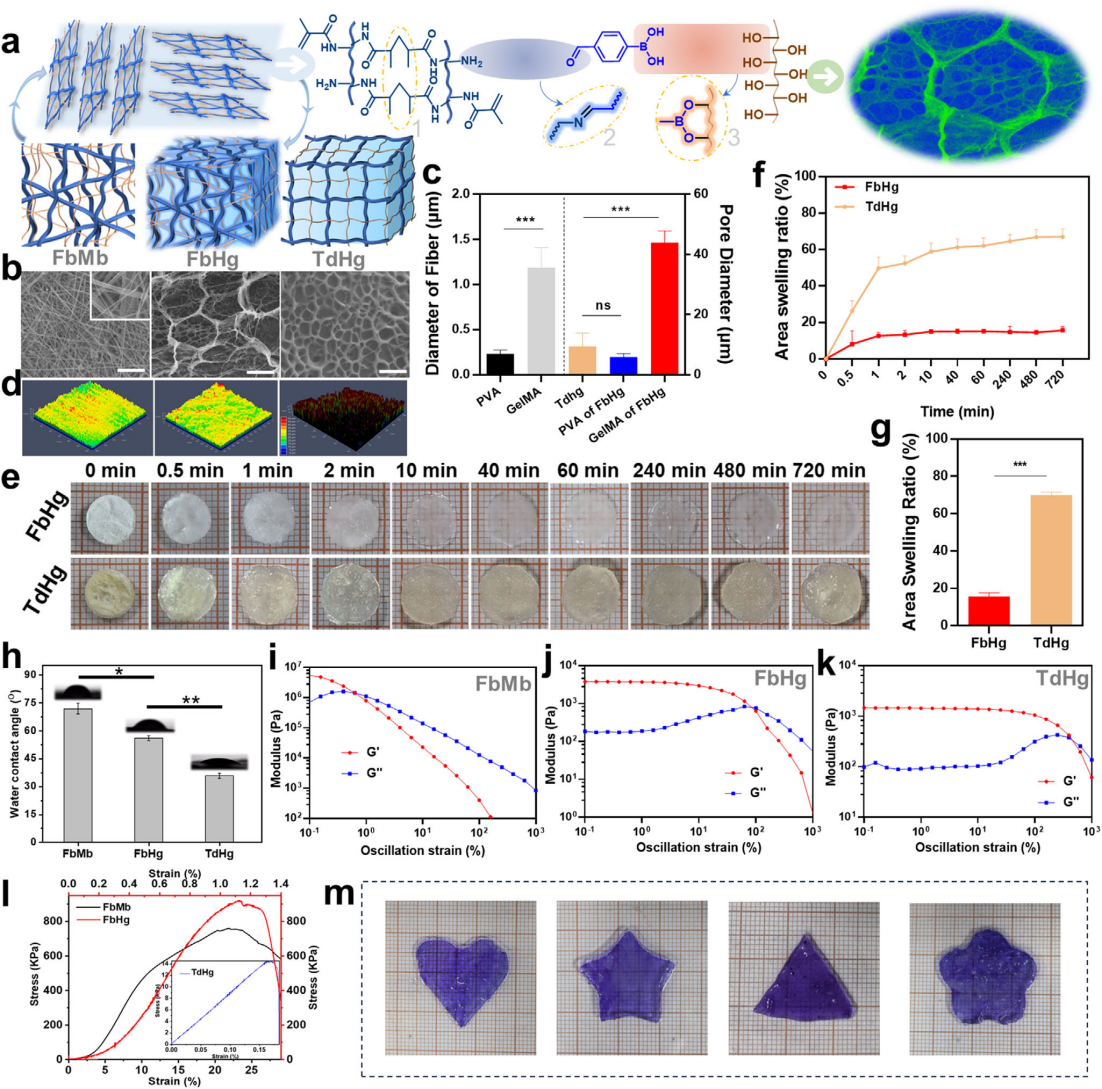
Physicochemical characteristics of the spatially heterogeneous hydrogel. a) Schematic diagram showing the formation of the fibrous membrane and hydrogel network. b) SEM microstructures of FbMb, FbHg, and TdHg (scale bar = 30 µm). c) Quantitative analysis of fiber diameters and hydrogel pore sizes. d) Surface microprofiles of the three materials. e–g) Qualitative and quantitative analyses of the swelling properties of FbHg and TdHg hydrogels. h) Water contact angles of the three materials. i–k) Rheological performance of different hydrogel components. l) Tensile strength of the fibrous membrane and two hydrogels. m) Versatile formation of FbHg into various shapes during the transition from fibrous membrane to hydrogel. *n* = 3, ***p < 0.001, **p < 0.01, *p < 0.05.

Hydrogels are among the most popular and representative biomaterials for tissue repair due to their controllable structure, performance, and functionality, as well as their similarity to biological tissues.^[^ ^12^, ^28^ ^]^ Generally, hydrogels can be tailored to form typical 3D network structures by modulating intermolecular and intramolecular interactions using various polymers, monomers, crosslinking agents, and synthetic strategies.^[^ ^29^, ^30^ ^]^ Herein, we employed a specialized technique to develop a spatially heterogeneous hydrogel system for treating infectious tissue injuries. Methacrylated gelatin (GelMa), the photoinitiator lithium phenyl (2,4,6‐trimethylbenzoyl) phosphinate (LAP), and the crosslinking molecule 4‐formylphenylboronic acid (4FA) were blended as one component. Polyvinyl alcohol (PVA) and POM served as another component. Using a dual‐channel co‐electrospinning technique, a composite fibrous membrane (FbMb) was prepared. Subsequently, under immersion conditions and through secondary photo‐crosslinking, the formation of a fiber hydrogel (FbHg) with a stable spatially heterogeneous network structure was achieved by establishing chemical bonds both within and between fibers. The formation of this specific hydrogel network primarily involves chemical interactions among molecular chains in the interwoven fibers: ① Covalent crosslinking of unsaturated double bonds in GelMa fibers, ② Imine bonds formed between amino groups in GelMa fibers and aldehyde groups in 4FA, ③ Boronic ester bonds formed between vicinal diols in PVA fibers and boronic acid groups in 4FA. In aqueous media, these chemical bonds reconstruct the internal 3D network, forming a gel state with spatial heterogeneity (FbHg) (**Figure** **2**a). To explore the differences between the hydrogel prepared using this process and conventional hydrogels, a traditional hydrogel (TdHg) was fabricated using the same components for comparison. Micromorphological images show that GelMa fibers and PVA fibers are regularly interwoven to form a typical fibrous membrane structure. Following secondary crosslinking within and between fibers, FbHg undergoes structural transformation and reorganization, forming a spatially heterogeneous 3D hydrogel network with varying pore sizes (Figure 2b,c). This significantly differs from the homogeneously distributed network structure of traditional hydrogels. The micro‐topography and surface roughness of the three samples also show notable differences (Figure 2d; Figure S1, Supporting Information). As shown in Figure 2e, the unordered stacking of fibers in FbMb results in a completely opaque state. As crosslinking progresses and the 3D network structure reconstructs, the transparency of the resulting hydrogel gradually increases. Moreover, as hydrogels are characterized by their high‐water content, TdHg exhibits significant volumetric swelling, which might cause interfacial mismatches with the damaged area when applied as a tissue repair material, potentially hindering the repair and regeneration process. The 3D network of FbHg, preformed from micro/nanoscale fibers and interwoven arrangements, restricts swelling effectively due to its dense internal network structure in conjunction with increased crosslinking (Figure 2e–g; Figure S2, Supporting Information). Therefore, FbHg demonstrates significant anti‐swelling properties, effectively avoiding excessive swelling when applied as a tissue repair material. Water contact angle analysis (Figure 2h) shows that TdHg has higher hydrophilicity, making it more prone to swelling, consistent with the observed swelling results. Rheological testing results (Figure 2i–k; Figures S3–S5, Supporting Information) reveal that the storage modulus (𝐺′) of both hydrogels exhibits typical elastic enhancement behavior, while loss modulus (𝐺′′) shows an initial increase followed by a decrease with increasing frequency, reflecting stronger viscous behavior at intermediate frequencies and reduced viscous effects at higher frequencies. Notably, FbHg exhibits stronger elasticity at high frequencies, potentially attributed to the transition of its internal structure from fibers to a 3D network, which offers advantages in tissue repair applications. Additionally, both 𝐺′ and 𝐺′′ remain stable within a low strain range, indicating a linear viscoelastic region. However, as strain increases, the storage modulus declines significantly, a typical characteristic of hydrogel yielding under high strain. During the transformation from fibrous membranes to hydrogels, FbHg undergoes multiple hierarchical crosslinking and structural rearrangements, resulting in significantly enhanced mechanical strength and thermal stability compared to TdHg (Figure 2l;Figures S6 and S7, Supporting Information). This trend is particularly evident for the specific components used in this study. FbHg maintains stability under physiological conditions, which is crucial for the development of biomedical materials. Furthermore, the flexibility of the fibrous membrane‐to‐hydrogel transition allows for various morphologies (Figure 2m), laying the foundation for diverse potential applications.

**Figure 3:**
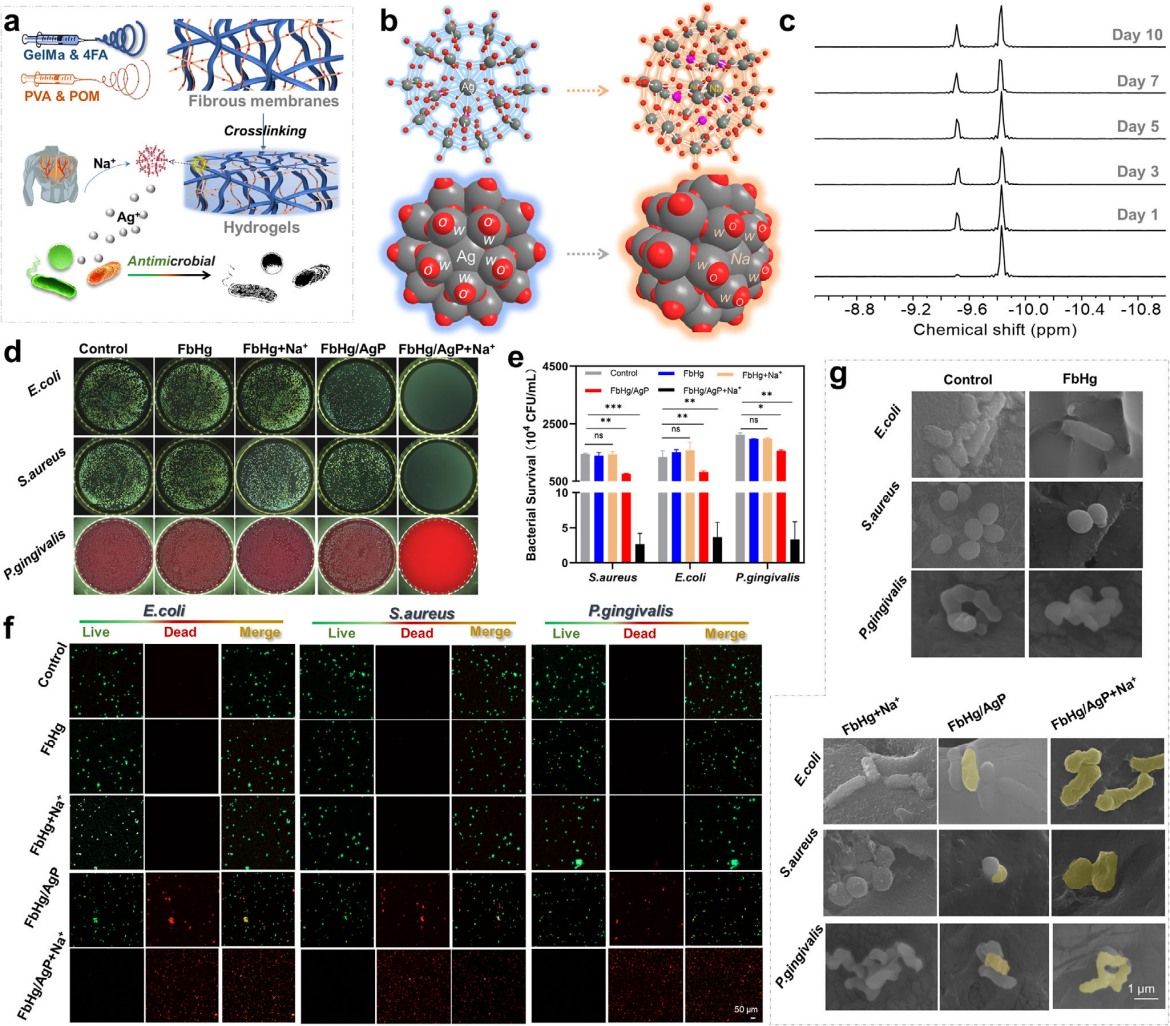
In vitro antibacterial effects of the hydrogel system. a) Schematic illustration of bioresponsive factor release from the hydrogel. b) Molecular simulation of AgP5W30 transforming into NaP5W30 under Na⁺ induction. c) Time‐gradient ^3^¹P NMR spectra of Ag⁺ release induced by Na⁺. d,e) Bacterial plates and colony counts of *E. coli, S. aureus*, and *P. gingivalis* under different sample treatments. f,g) Fluorescent live/dead staining and SEM microstructures of bacteria treated with different hydrogels. *n* = 3, ***p < 0.001, **p < 0.01, *p < 0.05.

One of the critical challenges in repairing infectious tissue injuries is the persistent proliferation and invasion of bacteria, making bacterial inhibition and eradication essential during treatment. Therefore, the development of tissue repair materials must prioritize antibacterial functionality. To address the limitations of traditional antibiotics, particularly the emergence of bacterial resistance, this study adopts silver as an antibacterial component due to its broad‐spectrum efficacy and extensive application potential. However, studies have revealed that prolonged exposure of silver in specific physiological environments can result in bacterial aggregation and encapsulation, significantly reducing its antibacterial performance.^[^ ^19^ ^]^ To tackle this issue, we introduced a polyoxometalate (POM)‐based silver ion compound, AgP_5_W_30_ (AgP), as the antibacterial agent. The POM framework naturally protects Ag^+^ from encapsulation by anions and proteins. Interestingly, the abundance of Na^+^ in biological environments can displace Ag^+^ from AgP, thereby enabling its antibacterial activity. Na⁺ are abundant in biological fluids, particularly in plasma and interstitial fluid, where they constitute over 90% of extracellular cations, which is sufficient to mediate Ag+ release in vivo. Moreover, the external chemical sites of POM can readily form hydrogen bonds with the hydroxyl groups in PVA, stabilizing the POM within the initial fiber structure and hydrogel network, facilitating subsequent biomedical applications (**Figure** **3**a). POM is pre‐loaded into polymer fibers and subsequently undergo covalent crosslinking to form the hydrogel network, ensuring that the active components are more uniformly distributed within the fibers rather than simply diffusing into a homogeneous hydrogel matrix. Furthermore, this structural characteristic plays a crucial role in regulating the release behavior of components with different properties and functions, allowing them to exert functions in a more favorable microenvironment. It is worth noting that the spatially heterogeneous structure of hydrogel system allows therapeutic components with different sizes and physicochemical properties to be preferentially distributed in suitable micro‐regions within the hydrogel matrix. This arrangement enhances functional stability. The mechanism of Na^+^‐induced Ag^+^ displacement in POM is attributed to the dehydration energy of cations, which dictates the encapsulation and release capabilities of the Preyssler‐type POM.^[^ ^31^, ^32^ ^]^ Na^+^, with relatively low dehydration energy,^[^ ^33^, ^34^ ^]^ can replace Ag^+^ under mild conditions (Figure 3b). This process was further investigated using ^13^P nuclear magnetic resonance (NMR) spectroscopy to track the Na^+^‐triggered release of Ag^+^ over time. The intensity of the characteristic peak at −10.4 ppm gradually decreased, while the peak at −10.1 ppm corresponding to [NaP_5_W_30_O_110_]^14−^ increased, confirming the displacement of Ag^+^ by Na^+^ and its controlled release (Figure 3c; Figure S8, Supporting Information). To evaluate the antibacterial efficacy of this system, comprehensive in vitro experiments were conducted against *Escherichia coli* (*E. coli*), *Staphylococcus aureus* (*S. aureus*), and *Porphyromonas gingivalis* (*P. g*). As shown in Figure 3d,e, the hydrogel alone exhibited no antibacterial activity. In contrast, the Ag^+^‐loaded POM (FbHg/AgP) group demonstrated stable incorporation of POM without Ag+ release in the absence of Na+. Upon exposure to exogenous Na^+^ (FbHg/AgP+Na^+^), Ag^+^ was effectively released, resulting in significant antibacterial activity. Live/dead fluorescence staining revealed that the control group exhibited a predominance of live bacteria (green), while the FbHg/AgP+Na^+^ group showed a substantial reduction in live bacteria and an increase in dead bacteria (red), indicating potent antibacterial effects (Figure 3f). Observations of bacterial morphology (Figure 3g) further confirmed the bactericidal action of Ag^+^, consistent with the colony count and fluorescence staining results. These findings validate the feasibility and effectiveness of the FbHg/AgP+Na^+^ hydrogel system as a broad‐spectrum antibacterial platform, offering promising prospects for treating infectious tissue injuries.

**Figure 4:**
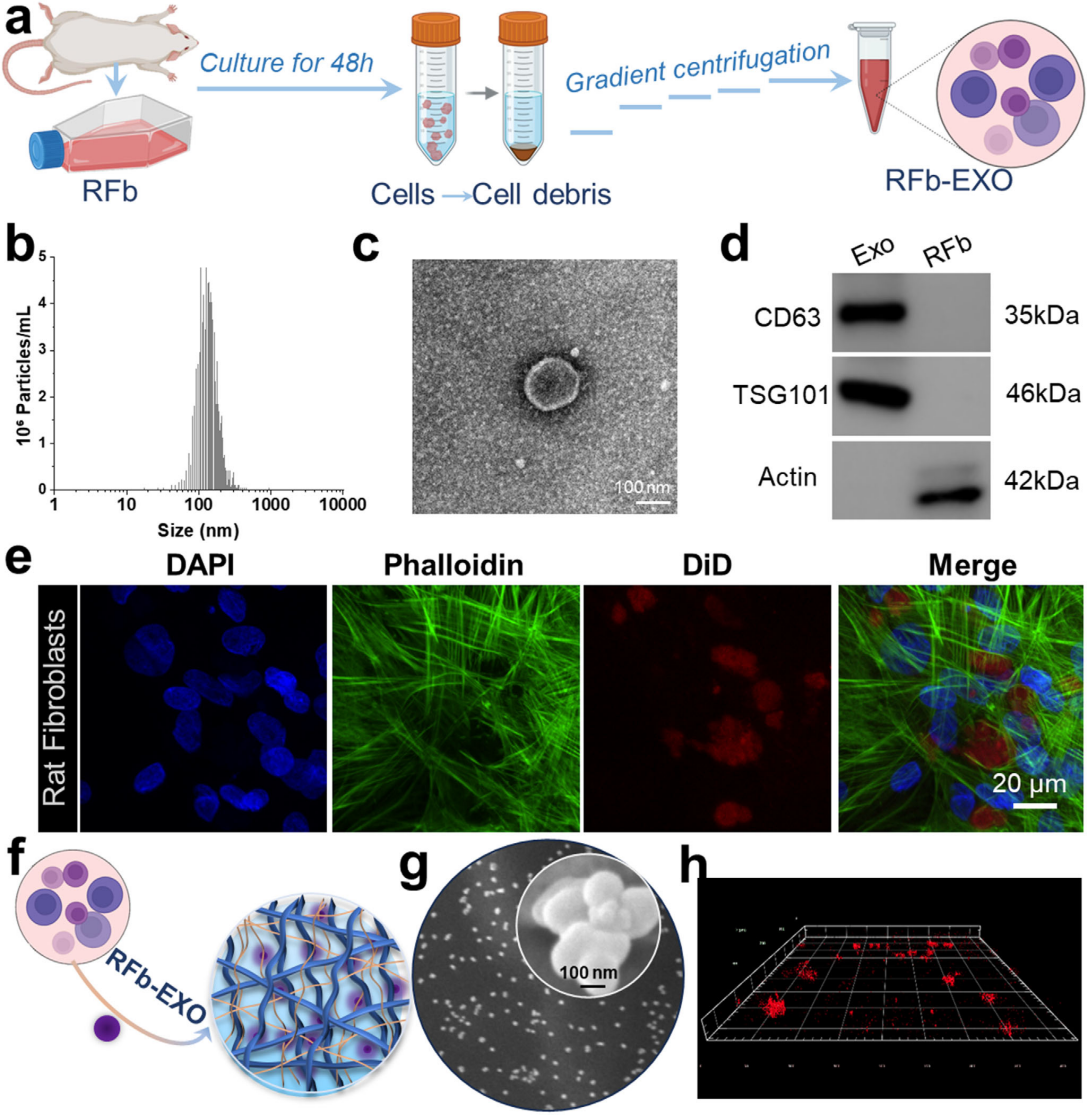
Isolation, characterization, and loading of exosomes. a) Schematic illustration of exosome isolation and extraction. b–d) Characterization of exosomes (size distribution, morphology, and protein markers). e) Confocal microscopy images of cellular uptake of exosomes. f) Schematic representation of exosome loading into hydrogels. g) SEM micromorphology of exosomes. h) 3D fluorescence imaging of hydrogels labeled with fluorescent exosomes.

An ideal biomaterial system for repairing infectious tissue injuries must combine antibacterial properties with excellent biocompatibility and the ability to promote cell proliferation, differentiation, and tissue regeneration. In this study, a spatially heterogeneous hydrogel material with antibacterial functionality was designed. Its unique structure is well‐suited for loading diverse functional agents, such as drugs, proteins, and macromolecules. To further enhance the bioactivity of the material in the infected microenvironment, exosomes were introduced to synergize with Ag+ for promoting the repair and regeneration of infectious tissues. Exosomes, nanosized extracellular vesicles carrying mRNA, miRNA, and proteins, mediate intercellular communication, signal transduction, and metabolism, showing great potential in tissue repair.^[^ ^35^, ^36^, ^37^ ^]^To comprehensively investigate the role of exosomes in soft and hard tissue repair, exosomes were extracted from two cell types: rat fibroblasts (rFb) and bone marrow mesenchymal stem cells (BMSCs), yielding rFb‐Exo and BMSC‐Exo, respectively. As shown in **Figure** **4**a, exosomes were isolated using classical gradient centrifugation and characterized via size distribution analysis and transmission electron microscopy (TEM). The characteristic cup‐shaped morphology and size distribution ≈100 nm confirmed the identity of the exosomes (Figure 4b,c; Figure S9, Supporting Information). Western blot analysis revealed significantly increased expression of marker proteins CD63 and TSG101 in rFb‐Exo compared to rFb (Figure 4d; Figure S10, Supporting Information), further validating successful exosome isolation. Exosome uptake by cells was assessed by culturing DiD‐labeled exosomes with cells, followed by observation via confocal laser scanning microscopy (CLSM). The results showed efficient internalization of exosomes, which were evenly distributed around the perinuclear region (Figure 4e; Figure S11, Supporting Information). Exosomes were then loaded into the hydrogel network by infiltration, enabling programmed migration within the hydrogel to exert bioactive functions. 3D confocal imaging and scanning electron microscopy (SEM) confirmed the distribution of exosomes within the hydrogel (Figure 4f–h; Figure S12, Supporting Information). The spatially heterogeneous structure of the hydrogel accommodates diverse loadings and releases,^[^ ^38^ ^]^ laying a foundation for the synergistic release of antibacterial and bioactive components (Figure 3c; Figure S13, Supporting Information) for future applications in controlled release and infectious tissue repair.

**Figure 5:**
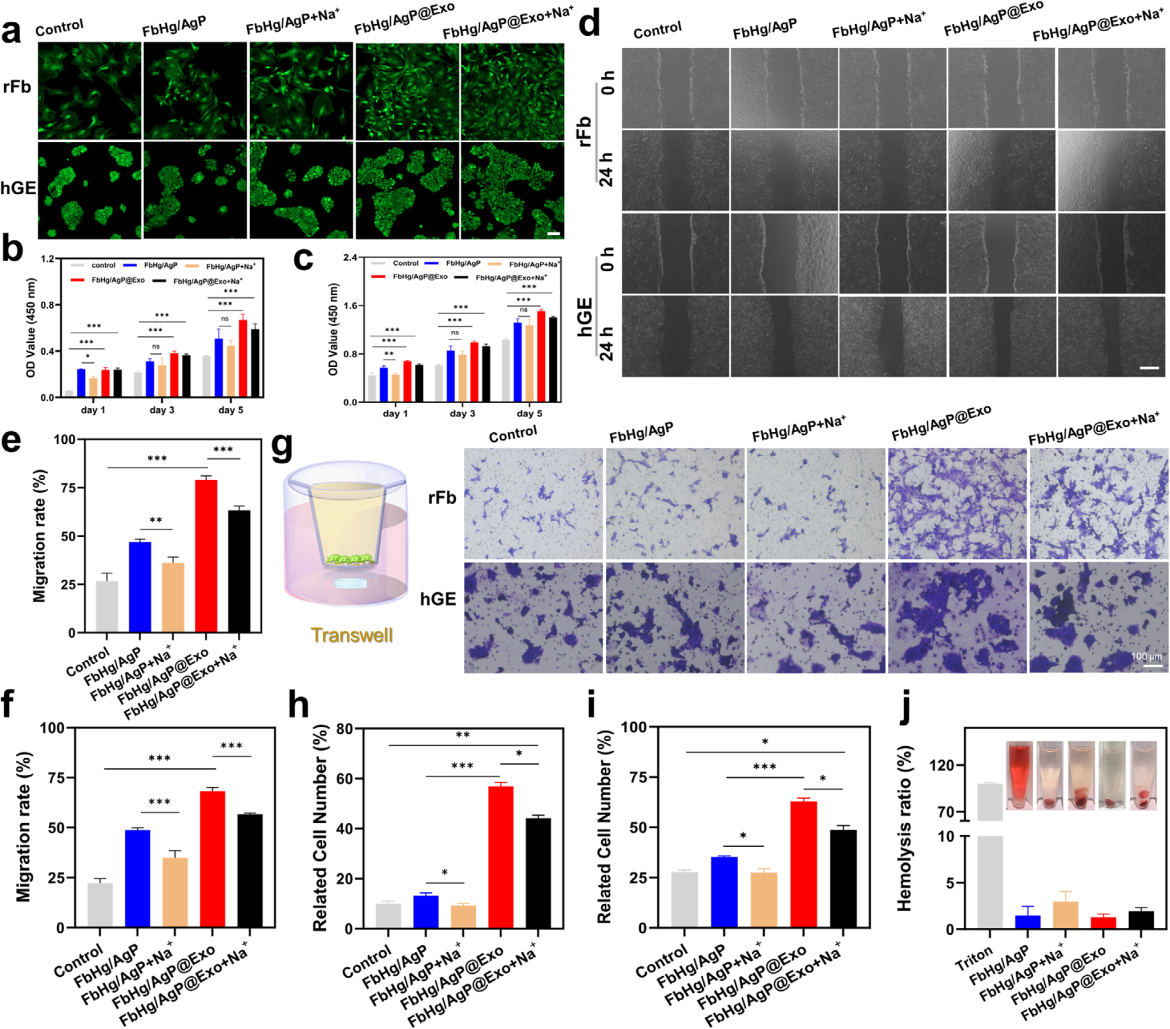
Effects of hydrogels on fibroblasts (rFb) and gingival epithelial cells (hGE). a) Live/dead staining of cells treated with different hydrogels. b,c) Quantitative analysis of cell viability using CCK‐8 for rFb and hGE cells. d–f) Representative images and quantitative analysis of cell scratch assay. g–i) Transwell assay evaluating the effects of different hydrogels on cells. j) The hemolysis rates of all hydrogel samples. *n* = 3, ***p < 0.001, **p < 0.01, *p < 0.05.

Two types of cells, rFb and human gingival epithelial cells (hGE), were selected to evaluate the in vitro biological effects of the exosome‐loaded hydrogel system. Initially, cell viability and cytotoxicity of the hydrogel system were assessed using live/dead staining and CCK8 assays (**Figure** **5**a–c). Compared to the control group, all hydrogel samples maintained good cell viability. On the first day, the introduction of Na⁺ had a certain impact on cell viability, which might be due to the fact that Na⁺ guided the release of Ag⁺. As cells proliferated and differentiated, no significant differences were observed among the hydrogel samples on day 3 and day 5. Overall, none of the components in the hydrogel system exhibited cytotoxicity. As shown in the scratch wound healing assay results (Figure 5d–f), the GelMA‐containing FbHg hydrogel provided partial nutrients that supported cell growth, resulting in faster repair rates compared to the control group. The addition of exosomes further enhanced bioactivity, significantly promoting scratch closure. In addition to evaluating 2D wound healing, a transwell assay was used to investigate cell proliferation and differentiation in the vertical direction (Figure 5g–i). Consistent with the scratch assay results, exosome bioactivity significantly promoted cell proliferation and differentiation, highlighting its potential to aid the repair and regeneration of infected tissues. Blood compatibility is a critical safety parameter for tissue repair materials, particularly those that come into contact with blood, as it determines their clinical applicability. Hemolysis analysis (Figure 5j) demonstrated that the hemolysis rates of all hydrogel samples were significantly lower than those of the Triton group and remained below the 5% threshold required for clinical applications.^[^ ^36^, ^37^ ^]^ These results confirm that the hydrogel system exhibits excellent blood compatibility, supporting its feasibility for practical biomedical applications.

**Figure 6:**
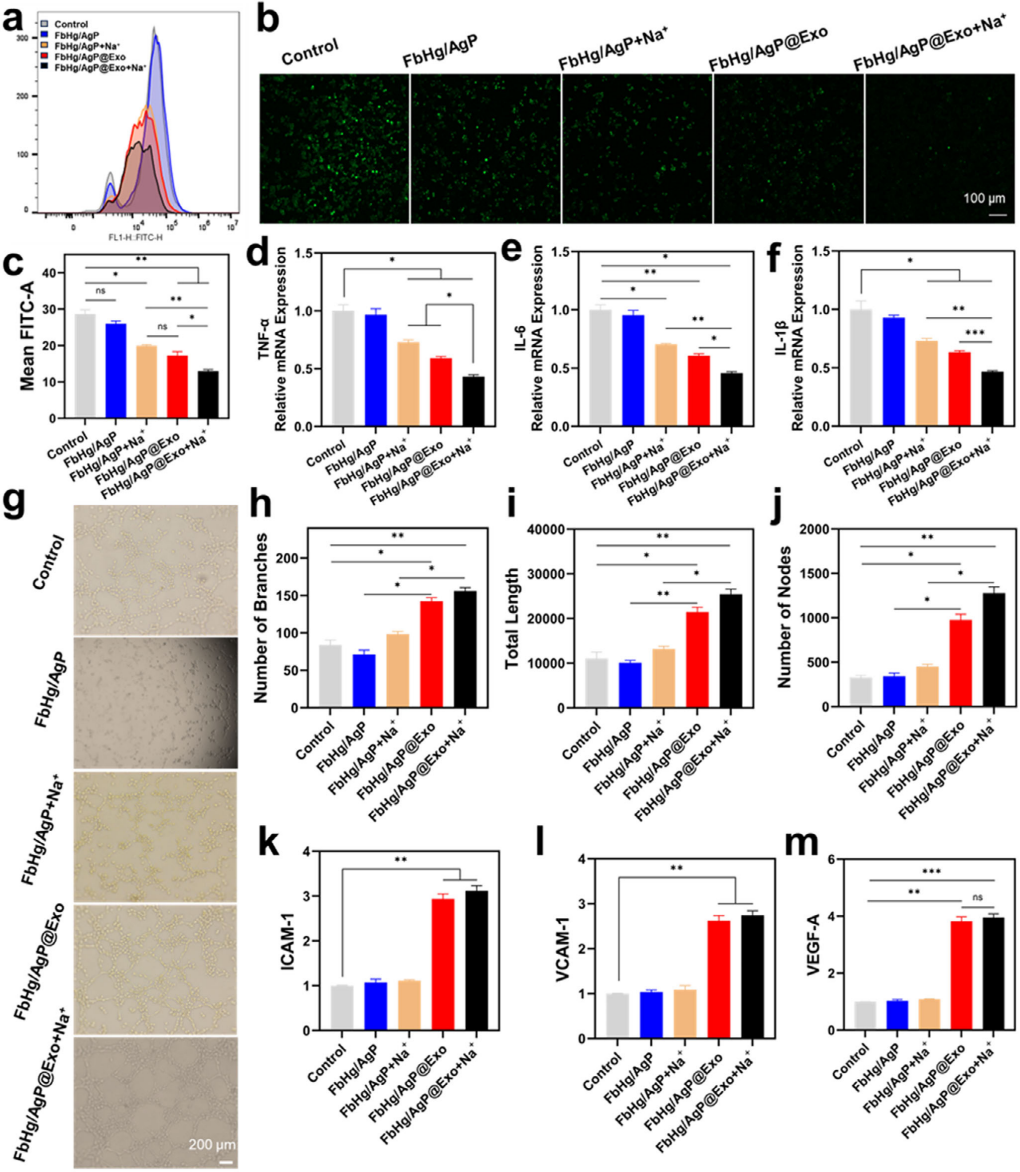
In vitro investigation of the bioactivity of exosomes. a) Intracellular ROS levels measured by flow cytometry. b,c) Fluorescent ROS staining under different hydrogel treatments. d–f) Quantitative qRT‐PCR analysis of TNF‐α, IL‐6, and IL‐1β expression in cells. g) Bright‐field images of tube formation assays. h–j) Quantitative analysis of network branching, total length, and nodes of tube formation. k–m) Expression of ICAM‐1, VCAM‐1, and VEGF‐A genes in cells. *n* = 3, ***p < 0.001, **p < 0.01, *p < 0.05.

High oxidative stress in infectious microenvironments critically affects chronic inflammation and tissue repair processes. Impaired transitions from the inflammatory to proliferative phases are primarily driven by reactive oxygen species (ROS) and pro‐inflammatory chemokines induced by resistant bacterial infections.^[^ ^36^, ^39^, ^40^ ^]^ To mitigate high ROS levels, the repair material was designed to synergize exosome bioactivity with hydrogel components to suppress ROS in inflammatory microenvironments. As shown in **Figure** **6**a–c, under identical magnifications using confocal microscopy, FbHg/AgP+Na^+^, FbHg/Agp@Exo, and FbHg/AgP@Exo+Na^+^ all exhibited reduced intracellular ROS levels compared to the control group, indicating their ability to scavenge ROS and decrease ROS accumulation. The release of Ag^+^ from POM within the hydrogel contributed to ROS clearance, while the introduction of exosomes further enhanced this antioxidant capacity. Flow cytometry analysis corroborated the confocal results, demonstrating significant reductions in intracellular ROS levels across the three experimental groups. These findings collectively indicate that the FbHg/AgP@Exo+Na^+^ hydrogel system exhibits potent antioxidant effects, effectively suppressing ROS in infectious microenvironments and facilitating tissue repair. Herein, the combined action of Ag⁺ ions and exosomes significantly enhanced the capacity to scavenge reactive oxygen species (ROS). Ag⁺ ions, as metal ions with unique redox properties, are capable of directly neutralizing ROS, thereby reducing their accumulation. Exosomes, on the other hand, carry a variety of bioactive molecules, including antioxidant enzymes, small RNAs (miRNAs), and antioxidant proteins. These molecules can be delivered to recipient cells via the targeting function of exosomes, thereby enhancing the cells' antioxidant capacity.^[^ ^35^, ^36^ ^]^ pro‐inflammatory cytokines such as TNF‐α, IL‐6, and IL‐1β, which are triggered by infectious microenvironments, exacerbate the chronic inflammation cycle. qRT‐PCR analysis of pro‐inflammatory cytokine expression (Figure 6d–f) revealed a significant reduction in TNF‐α, IL‐6, and IL‐1β levels in the FbHg/AgP@Exo+Na^+^ group, indicating that the incorporation of exosomes effectively mitigates inflammation by suppressing cytokine expression, thereby fostering tissue repair and regeneration. Angiogenesis is a vital indicator of tissue repair, as it supplies nutrients and oxygen to critical cells during the repair process. The impact of the hydrogel system on angiogenesis was assessed in vitro. As shown in Figure 6g, the exosome‐containing hydrogel group exhibited significant vascular network formation and branching compared to the control group. Quantitative analysis further confirmed increased numbers of vascular tubes, nodes, and total network length in the exosome‐loaded groups (Figure 6h–j). Additionally, the expression levels of angiogenic‐related genes, including intercellular adhesion molecule‐1 (ICAM‐1), vascular cell adhesion molecule‐1 (VCAM‐1), and vascular endothelial growth factor‐A (VEGF‐A), were markedly upregulated in the FbHg/AgP@Exo+Na^+^ group (Figure 6k–m). Exosomes, enriched with various bioactive components such as proteins and miRNAs, significantly enhanced the expression of angiogenesis‐related factors.^[^ ^41^ ^]^ This capability not only promoted the formation of vascular networks but also compensated for oxygen and nutrient deficiencies in the damaged microenvironment caused by infectious tissue injury, providing a robust potential to accelerate tissue repair and regeneration.^[^ ^42^, ^43^ ^]^

**Figure 7:**
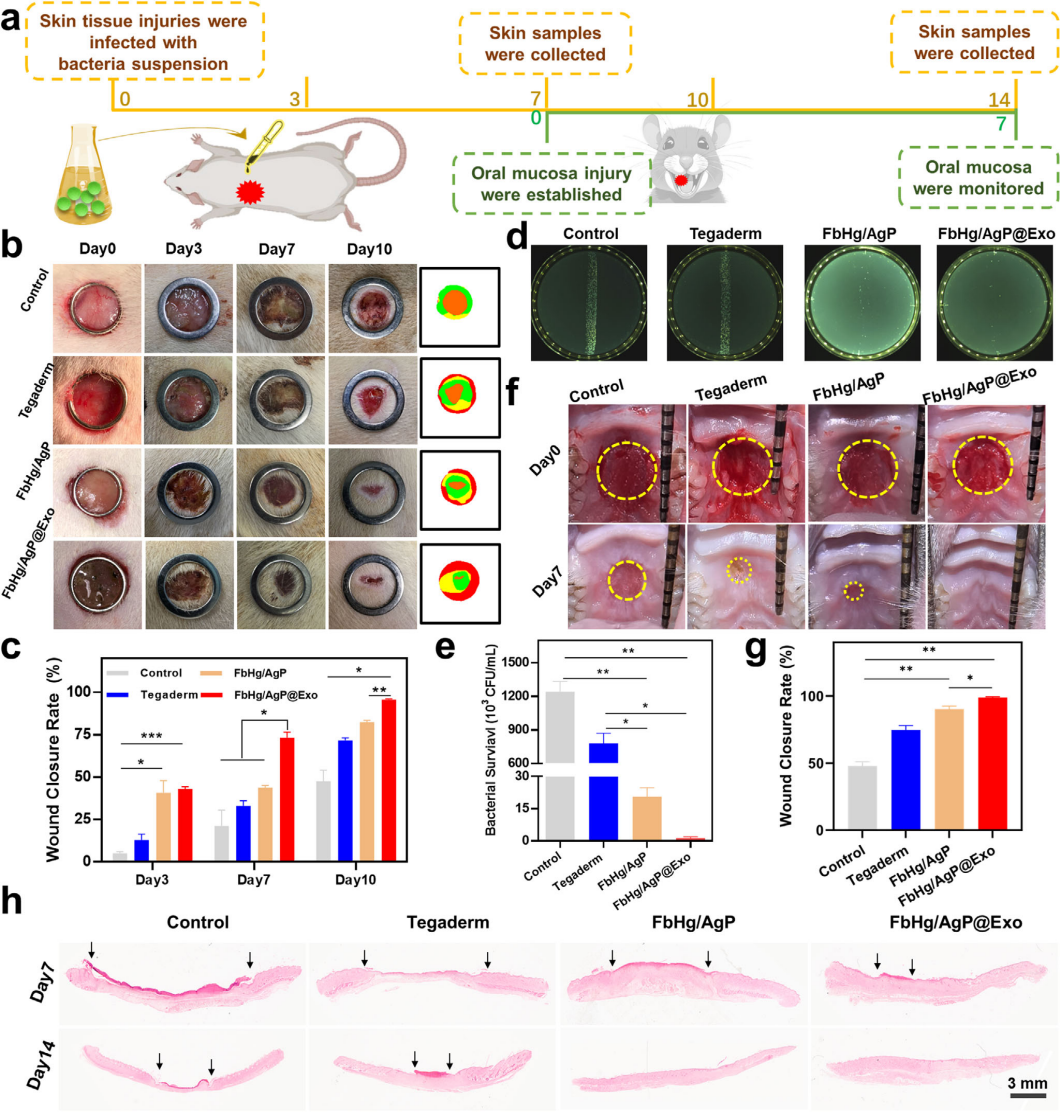
Application of the POM‐exosome hydrogel system for treating infectious soft tissue injuries. a) Modeling process of the bacterial infection injury model. b,c) Macroscopic images and quantitative repair rate analysis of infection sites treated with different hydrogels. d,e) Bacterial plate assays and quantitative analysis of infection sites. f,g) Macro photograph and quantified healing rates of oral mucosal injuries treated with hydrogels. h) H&E staining images of infection sites under different treatments. *n* = 3, ***p < 0.001, **p < 0.01, *p < 0.05.

The mechanisms underlying the treatment challenges of infectious tissue injuries are complex, involving multidrug‐resistant bacterial infections, chronic inflammation, vascular damage, and oxidative stress. In this study, an animal model of exogenous bacterial‐infected tissue injury was constructed to evaluate the effects of the designed therapeutic system on infectious tissue injuries. To validate the universality of the system, three experimental animal models were established, representing soft tissue injuries (skin and mucosa) and hard tissue injuries (bone). All animal experiments were approved and supervised by the Animal Ethics Committee of Sun Yat‐sen University (SYSU‐IACUC‐2024‐B1635, SYSU‐IACUC‐2024‐B1583, SYSU‐IACUC‐2024‐B1634). The specific experiments were carried out according to a pre‐designed protocol (**Figure** **7**a). Tissue injuries were induced on the backs and oral mucosa of rats, and bacterial infections were introduced to the injury sites. The animals were divided into four groups to assess the differences in repair efficacy for infectious tissue injuries. Macroscopic observations of the soft tissue injury sites and the repair processes showed that the injured areas in all groups reduced over time, with the FbHg/AgP@Exo group exhibiting the fastest healing rate compared to other groups (Figure 7b,c; Figure S14, Supporting Information). In the untreated control group, the bacterial‐infected injury sites developed typical purulent symptoms, and without intervention, the infected tissues struggled to self‐heal. Plate culturing of fluids collected in situ from the infected sites revealed significantly reduced bacterial counts in groups containing POM (Figure 7d,e), attributed to the sodium‐ion‐induced release of antibacterial silver ions. Additionally, the results from mucosal tissue injury repair demonstrated the superior efficacy of the FbHg/AgP@Exo group compared to other groups, with significant differences observed (Figure 7f,g). These results are due to the combined effect of antibacterial Ag^+^ released by POM, which binds to biologically active exosomes.

**Figure 8:**
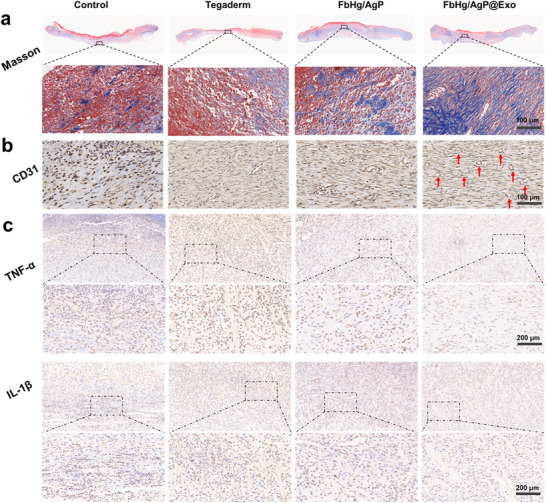
Histological evaluation of infectious soft tissue injuries. a) Masson's trichrome staining of injury site sections on postoperative day 7. b) CD31 immunohistochemical staining of tissue sections on day 14. c) TNF‐α and IL‐1β immunohistochemical staining of injury sites on postoperative day 7. Dashed black boxes in the upper images are magnified below.

H&E and Masson's trichrome staining were employed to evaluate the histological condition of infectious tissue injuries. Both the negative and positive control groups exhibited poor healing in the defect areas. In contrast, the FbHg/AgP@Exo group demonstrated significantly faster granulation tissue formation, the thickest and most complete epidermal differentiation, and the most pronounced formation of new tissue (Figure 7h). Collagen formation and distribution, critical indicators of dermal regeneration, showed significant differences among groups. As anticipated, while all groups exhibited varying degrees of epithelialization and collagen deposition, the collagen in the control groups was loosely and disorganizedly distributed. Conversely, the FbHg/AgP@Exo group displayed markedly increased collagen deposition, characterized by dense and orderly aligned collagen fibers (**Figure** **8**a; Figure S15, Supporting Information), indicating a favorable trend for promoting tissue regeneration. In infectious tissue injuries, the damaged vascular system exacerbates the local microenvironment. Neovascularization, a key factor in tissue repair, provides the nutrients and oxygen essential for regeneration. CD31 immunohistochemical staining was used to assess angiogenesis (Figure 8b). The control and Tegaderm groups exhibited low CD31 expression with incomplete endothelial integration, while groups containing Exos showed significantly higher expression of related proteins, with the most abundant and uniformly distributed neovascularization (Figure S16, Supporting Information). Finally, immunohistochemical staining was performed to evaluate the expression of pro‐inflammatory cytokines, interleukin‐1β (IL‐1β) and tumor necrosis factor‐α (TNF‐α) (Figure 8c; Figure S17, Supporting Information). The control group showed markedly higher positive expression of IL‐1β and TNF‐α, indicating a strong inflammatory response in the injury area. Compared to other groups, the FbHg/AgP@Exo group exhibited significantly reduced expression of these cytokines, facilitating the transition from the inflammatory phase to the proliferation phase. This result aligns with the in vitro findings. The synergistic effects of exosomal bioactivity and antibacterial functionality offer a promising strategy for developing clinical therapeutic approaches.

**Figure 9:**
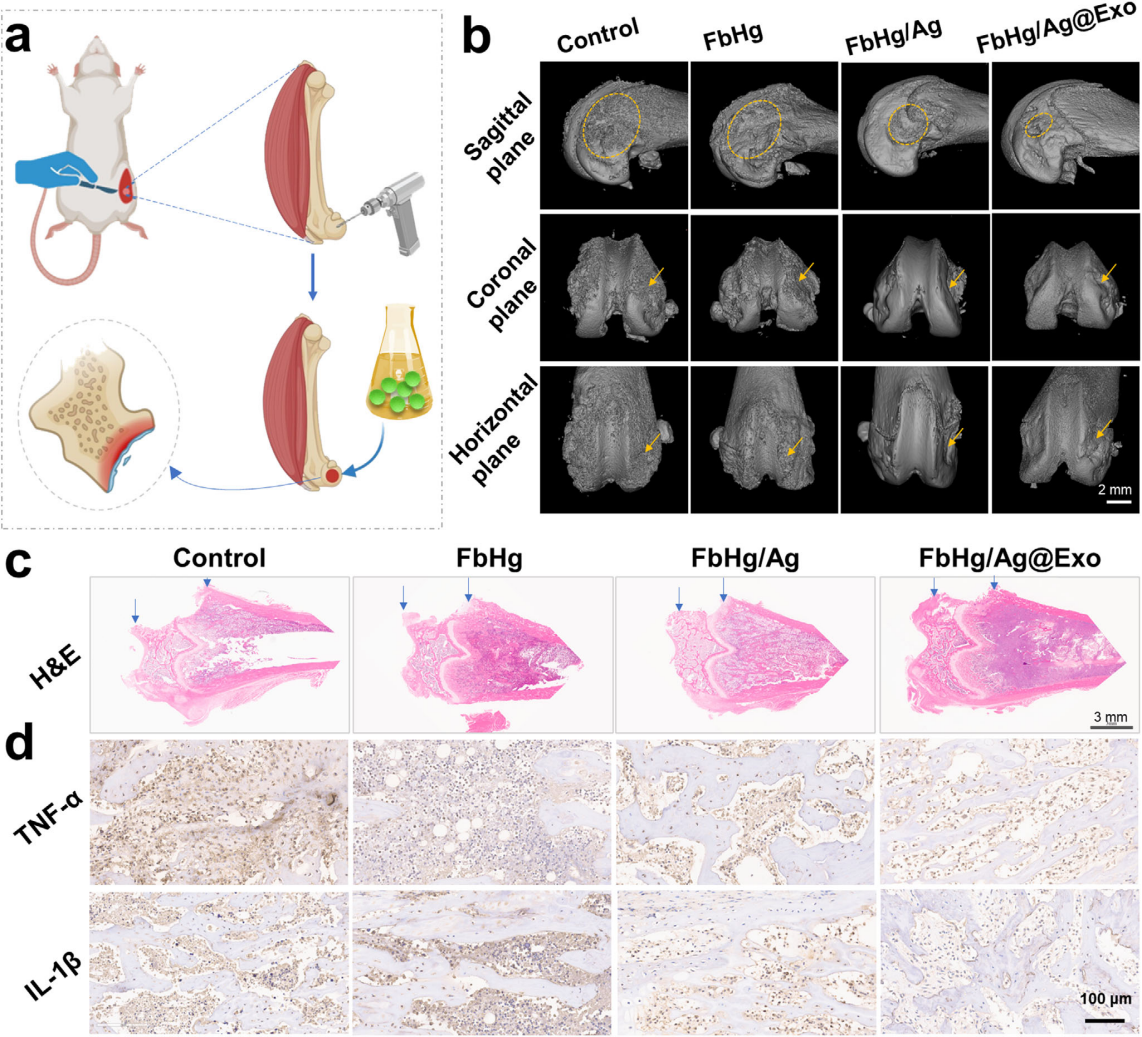
Application of the POM‐exosome hydrogel system for treating infectious hard tissue injuries (4 weeks post‐operation). a) Modeling process of the bacterial infection‐induced bone injury model. b) Micro‐CT 3D reconstruction images of bone defects treated with different hydrogels. c) H&E staining images of infected bone treated with various hydrogels. d) Immunohistochemical staining of TNF‐α and IL‐1β in bone defect sites.

The efficacy of the material system for hard tissue repair and regeneration was further validated using an infectious tibia defect model (**Figure** **9**a). Micro‐CT 3D reconstruction analysis revealed significant bone destruction and poor morphology in the control group, which were exacerbated by bacterial invasion (Figure 9b; Figure S18, Supporting Information). In contrast, the FbHg/AgP@Exo group exhibited significantly higher bone density than other groups, with the bone volume to tissue volume ratio (BV/TV) consistently superior at different time points, indicating the formation of abundant new bone tissue. H&E staining showed that the control group exhibited no obvious healing, and the defect area appeared to enlarge (Figure 9c). The arrows indicate the femoral medial condyle defect in the H&E‐stained sections. In the FbHg/AgP@Exo group, the defect was minimal, with only a shallow saucer‐shaped lesion. The FbHg/AgP group showed incomplete healing with a concave defect, while the FbHg group exhibited a larger defect. In the control group, the defect was significantly enlarged beyond the initial injury due to bacterial invasion and tissue degradation. Moreover, immunohistochemical staining of inflammatory cytokines (Figure 9d; Figure S19, Supporting Information) showed that TNF‐α and IL‐1β positive staining was significantly reduced in the FbHg/AgP@Exo‐treated tissues compared to the control group, consistent with the in vitro anti‐inflammatory results. This effect was attributed to the early antibacterial intervention of Na⁺‐triggered Ag⁺ release and the anti‐inflammatory capacity of exosomes. As previously mentioned, one of the defining features of infectious tissue injuries is bacterial invasion. Suppressing bacterial or microbial infections during the early stages of repair is critical for successful tissue regeneration. The Na⁺‐induced release of Ag⁺ ions from the polyoxometalate matrix provided exceptional antibacterial functionality, contributing significantly to early‐stage infection management. The incorporation of Ag⁺ into POM improved biocompatibility and prevented the decline in antibacterial activity associated with prolonged exposure. The bioactivity of exosomes was a key factor during the mid‐to‐late stages of treatment. In summary, the FbHg/AgP@Exo hydrogel system presents an effective solution to the challenges of treating infectious tissue injuries in clinical applications. The combined functionalities of Ag⁺ and exosomes within the hydrogel target infection‐related tissue damage, while the endogenous Na⁺‐induced release of Ag⁺ ions ensure superior early‐stage bacterial inhibition. This advanced system holds great promise for addressing the complexities of infectious tissue injury repair and regeneration. It is worth noting that exosomes are promising for clinical applications due to their natural origin and low immunogenicity.^[^ ^44^, ^45^, ^46^, ^47^ ^]^ However, challenges in large‐scale production, standardization, and quality control hinder their translation.^[^ ^45^, ^46^ ^]^ Ensuring consistency and stability remains difficult, requiring further optimization to improve manufacturability and clinical adoption.^[^ ^46^ ^]^

## 3 Conclusion

This study developed a therapeutic hydrogel system based on nanoscale polyoxometalates (POMs) for the treatment of infectious tissue injuries. By encapsulating Ag^+^ in Preyssler‐type POM and incorporating it with PVA and GelMa to construct a multi‐scale spatially heterogeneous hydrogel, the system effectively protected Ag^+^ and enabled its precise release upon Na^+^ induction. The dual protection by the hydrogel and POM prevented the loss of antibacterial activity in physiological environments while maintaining excellent biocompatibility. Additionally, the specialized fabrication process of the hydrogel improved drug dispersibility, enhanced stability, and mitigated structural defects commonly associated with traditional hydrogels. Leveraging the combined effects of exosomes, the system demonstrated excellent antibacterial efficacy and tissue repair capacity in both in vitro and in vivo experiments, providing a novel strategy for treating infectious tissue injuries with significant clinical potential.

## Conflict of Interest

The authors declare no conflict of interest.

## Supporting information



Supporting Information

## Data Availability

The data that support the findings of this study are available from the corresponding author upon reasonable request.
