# Supplementary material for: A Bio‐Responsive Hydrogel with Spatially Heterogeneous Structure for Treating Infectious Tissue Injuries
Source: Adv Sci (Weinh). 2025 Apr 28;12(23):2500088. doi: 10.1002/advs.202500088 (PMC12199478; doi:10.1002/advs.202500088)
Supplement: Supplementary file 1 — Supporting Information [file ADVS-12-2500088-s001.docx]

**Supporting Information**

**A Bio-Responsive Hydrogel with Spatially Heterogeneous Structure for Treating Infectious Tissue Injuries**

*Zongtai Li, Tao Yang, Xiaolei Li, Panchao Yin, Bo Yang, Dongying Li, Yan Wang*, Wei Teng*, Qianqian Yu*, Weichang Li**

**Synthesis of GelMa：**Gelatin methacrylate (GelMa) was synthesized following our previous study^[1]^. Briefly, gelatin was dissolved in deionized water at 50°C to prepare a 10% (w/v) solution. Methacrylic anhydride (MA) was gradually added dropwise at a volume ratio of approximately 8%, and the solution was stirred continuously for 6 hours. During the reaction, saturated sodium bicarbonate solution was used to stabilize the pH. Excess solvent was added to terminate the reaction, and the product was freeze-dried to obtain GelMa for subsequent use.

**Synthesis of Polyoxometalates (POMs)：**Sodium tungstate dihydrate (Na₂WO₄·H₂O) was dissolved in water, followed by the slow addition of phosphoric acid (H₃PO₄). The solution was transferred to a polytetrafluoroethylene (PTFE)-lined autoclave and reacted at 120°C for 12 hours. After the reaction, water and potassium chloride (KCl) were added to the pale-yellow solution, stirred until dissolved, and filtered. The precipitate was washed and recrystallized. The crystalline product was redissolved in water, and a silver nitrate (AgNO₃) solution was slowly added under heating. The mixture was placed in a PTFE-lined autoclave and reacted at 160°C for 24 hours. The solution was filtered to remove insoluble substances, and potassium nitrate (KNO₃) was added to precipitate a white solid. The precipitate was washed and recrystallized to obtain the final product, AgP_5_W_30_.

**Hydrogel Fabrication：**Solution A was prepared by dissolving PVA (or PVA with POM) in water. Solution B was prepared by dissolving GelMa, photoinitiator LAP, and crosslinking agent 4FA in hexafluoroisopropanol. Solutions A and B were loaded into separate syringes, and electrospinning with a dual-channel mode was used to produce fibrous membranes (FbMb) with interwoven layers of A and B. FbMb was then crosslinked sequentially in a wet state and under UV irradiation to reorganize intra- and inter-fiber molecular structures, resulting in a spatially heterogeneous hydrogel (FbHg) with a unique structure. For exosome loading, exosome solutions were infiltrated into the hydrogel system. For traditional hydrogel preparation, GelMa/4FA solutions were prepared by heating and prolonged stirring. GelMa/4FA, LAP, and PVA were mixed in an aqueous solution under UV irradiation to produce TdHg hydrogel as a control group.

**Physicochemical Characterization：**The fibrous membranes and hydrogels were freeze-dried in liquid nitrogen and characterized using scanning electron microscopy (SEM; FEI, USA). Fiber diameter and hydrogel pore size were measured with ImageJ software. Surface roughness parameters Sa (arithmetic mean height) and Sz (maximum height) were analyzed using a confocal laser scanning microscope (LSM700, Zeiss, Germany). Surface hydrophilicity and hydrophobicity were assessed by water contact angle measurements (JCY-1, KRÜSS, Germany). Rheological properties were measured with a rheometer (ARES-G2, TA Instruments, USA). Amplitude sweep analysis was conducted at a constant angular frequency of 1 Hz and a strain range of 0.1–1000%, while frequency sweep analysis was performed within a 0.1–100 rad/s angular frequency range at a constant strain of 0.1% in the linear viscoelastic region. Tensile strength was tested using a universal mechanical testing machine (Instron, USA) following the GB/T 528-2009 standard. Thermogravimetric analysis (TGA) was conducted on freeze-dried samples (METTLER TOLEDO, Switzerland) within a temperature range of 30–600°C at a heating rate of 10°C/min under a nitrogen atmosphere. Swelling ratio was calculated as Swelling Ratio (%)= (Wt-W_0_)/W_0_*100%, where W_0_ and W_t_ are the initial dry weight and the weight at different time, respectively. Anti-swelling performance was evaluated using pre-cut samples immersed in PBS buffer, with area changes quantified as Swelling rate (%) = (S_t_-S_0_)/S_0_*100, where S_0_​ and S_t_ are the initial area and the area at different time, respectively. At least three parallel samples were tested for all analyses.

**Silver Ion Release from POM:** To investigate silver ion release induced by ion exchange, AgP_5_W_30_ was dissolved in deuterium oxide (D₂O), and sodium chloride (NaCl) was added. The Na⁺ concentration in the experiment was intentionally reduced to allow for controlled and sustained Ag⁺ release. Ion exchange efficiency at different time points was assessed using ^31^P NMR spectroscopy. The ion exchange reaction ^[2,3]^ can be described as:

[AgP_5_W_30_] + [𝑁a+] → [𝑁aAgP_5_W_30_]

[𝑁aAgP_5_W_30_] → [𝑁aP_5_W_30_] + [𝐴g+]

**Release of Encapsulated Components in the Hydrogel System:** Due to the unique nanocage structure of POMs, simultaneous release of different components is currently challenging to determine. In this study, bioactive components were encapsulated within the hydrogel system. To investigate their release behavior, BSA was used as a model substance. The hydrogel system containing BSA was placed in PBS medium and incubated at 37°C with constant shaking. Samples were collected at specific time intervals, and the released BSA concentration was measured using UV spectrophotometry.

**Exploration of *In Vitro* Antibacterial Function:** *E. coli* (ATCC 8739) and *S. aureus* (ATCC 6538) were inoculated into liquid medium and cultured at 37°C under 5% CO₂. Bacterial suspensions were streaked onto solid medium, incubated overnight, and single colonies were transferred to fresh medium for shaking culture at 37°C.

*P. gingivalis* (ATCC 33277) was inoculated into liquid medium and cultured under strict anaerobic conditions at 37°C for 3–6 days until turbidity developed. The bacterial suspension was then streaked onto solid medium and incubated anaerobically to allow colony formation. Single colonies were transferred to fresh liquid medium for continued anaerobic culture.

A stock solution of hemin chloride in sodium hydroxide was prepared and mixed well, followed by the addition of vitamin K₁ solution and pH adjustment to 7.4. The solution was filtered to produce Component I. Component II was prepared by dissolving BHI powder, yeast extract, and L-cysteine hydrochloride in water and sterilized by autoclaving. Liquid medium was prepared by mixing Components I and II at a 1:100 ratio. For solid medium, nutrient agar powder was added to Component II, autoclaved, and combined with Component I and defibrinated sheep blood, which was allowed to solidify at room temperature.

Bacterial suspensions were diluted, added to sterile EP tubes, centrifuged, and the supernatant was discarded. The pellets were resuspended in sterile distilled water, and test samples were immersed in the bacterial suspension. For control groups, bacterial suspensions were centrifuged, resuspended in saline, and FbHg/AgP samples were immersed in the saline bacterial suspension. All were incubated at 37°C for 12 h, followed by PBS dilution. Dilutions were plated, cultured, and colonies were counted.

After bacterial treatment under the same conditions, the samples were incubated at 37°C for 12 h. The materials were collected, centrifuged, and stained with live/dead bacterial staining solution (SYTO 9 and PI). Samples were protected from light, centrifuged to remove excess dye, resuspended in sterile PBS, and observed using CLSM. Red fluorescence indicated dead bacteria, while green fluorescence indicated live bacteria.

Bacterial suspensions were diluted, resuspended in saline or sterile water, and inoculated onto the surface of test samples. After incubation at 37°C for 12 h, the samples were washed with sterile PBS, fixed with glutaraldehyde, dehydrated with gradient ethanol, freeze-dried, and examined under SEM to observe bacterial morphology on the material surface.

**Exosome Isolation and Characterization:** The back skin of 1–3-day-old neonatal rats was collected aseptically, rinsed with pre-cooled sterile PBS, and incubated with 0.25% trypsin/0.02% EDTA solution at 4°C for 12 h to separate the epidermis. The dermis was collected and digested with the same solution at 37°C with shaking. After centrifugation, the supernatant was discarded, and the tissue was resuspended in high-glucose DMEM containing 10% FBS and cultured at 37°C with 5% CO₂. Cells were passaged until 80–90% confluency, with the third passage used for experiments.

The femurs of 7–13-day-old neonatal rats were soaked in sterile PBS. Using PBS containing 2% FBS, the marrow cavity was flushed, and the contents were centrifuged. The pellet was resuspended in complete medium (DMEM/F-12 containing 10% FBS and 1% P/S) and cultured at 37°C with 5% CO₂ until the third passage.

Exosomes were isolated via ultracentrifugation. Conditioned medium from RFbs and BMSCs (3–5 passages) cultured in exosome-free medium was collected. After gradient centrifugation at 4°C, the supernatant was discarded, and the pellet was resuspended in sterile PBS. The suspension was filtered through a 0.22 μm filter to obtain sterile exosome suspension. Exosomes were deposited onto copper grids, stained with 2% uranyl acetate solution, and observed using TEM. Nanoparticle Tracking Analysis (NTA) was used to measure exosome size.

Exosome protein concentration was determined using a BCA kit. Protein standards were serially diluted, and BCA working solution was added to both standard and sample wells. After incubation at 37°C, absorbance at 562 nm was measured using a microplate reader, and protein concentrations were calculated.

For Western blot analysis, protein samples were denatured, separated by SDS-PAGE, and transferred to PVDF membranes. Membranes were blocked, incubated with primary antibodies overnight at 4°C, followed by secondary antibodies at room temperature for 1 h. After TBST washes, chemiluminescent detection reagents were added, and signals were imaged.

**Distribution of Exosomes in Hydrogel and Cellular Uptake Observation:** Exosomes were labeled with DiD, centrifuged to remove excess dye, and incorporated into the hydrogel system. Their distribution was observed and imaged using CLSM. Freeze-dried hydrogels containing exosomes were examined under SEM for exosome distribution.

DiD-labeled exosomes were incorporated into the hydrogel system and co-cultured with RFbs and BMSCs. Cells were fixed with paraformaldehyde, washed with PBS, and stained with phalloidin and DAPI. CLSM was used to observe and image cellular uptake.

**In Vitro Cellular Behavior:** RFbs and hGEs were seeded into culture plates and co-cultured with medium containing material components. At specific intervals, samples were collected for CCK-8 assays, and absorbance at 450 nm was measured using a microplate reader.

Cells were co-cultured with material components at 37°C, washed with PBS, and stained with live/dead cell staining solution under light protection. After washing, CLSM was used for observation.

Exponentially growing cells were digested with trypsin, resuspended, and counted. After cell attachment, a linear scratch was made with a pipette tip, washed with PBS, and cultured in serum-free medium. At specific time points, the scratch areas were imaged under a microscope, and the percentage of wound healing was calculated using ImageJ.

Exponentially growing cells were digested, resuspended in various media, and seeded in Transwell chambers. The lower chamber contained normal medium with 1% FBS. After incubation, the chambers were fixed, washed, and stained with crystal violet. Cell migration was observed and imaged under an inverted microscope, and cell numbers were counted using ImageJ.

**Hemolysis Assay:** Heparinized rat venous blood was centrifuged to obtain precipitated red blood cells, which were diluted with PBS according to their volume. The red blood cell suspension was mixed with solutions of different samples and incubated at 37°C. After incubation, the samples were centrifuged, and the layered state and color of the solutions were observed and recorded photographically. The supernatants from each group were collected, and the absorbance at 540 nm was measured using a microplate reader to calculate the hemolysis rate. The formula is as follows:

Hemolysis rate (%) = [(OD - OD₁) / (OD₂ - OD₁)] × 100%. Where: OD is the OD value of the supernatant from the sample group. OD₁ is the OD value of the PBS group supernatant. OD₂ is the OD value of the Triton X-100 group supernatant.

**2.12 Antioxidant Level Analysis:** Raw 264.7 cells were cultured until 70-80% confluence and then co-incubated with different sample groups. Cells were stimulated with hydrogen peroxide-treated extracts of the samples at 37°C. Afterward, the cells were digested with 0.25% trypsin, centrifuged to discard the supernatant, and resuspended with DCFH-DA diluted in serum-free medium. The mixture was incubated in the dark to ensure adequate probe-cell interaction, followed by washing with serum-free high-glucose DMEM to remove extracellular probes. Intracellular reactive oxygen species (ROS) fluorescence intensity was detected using a flow cytometer.

For confocal laser scanning microscopy (CLSM), Raw 264.7 cells were seeded and incubated with different media. After replacing the medium according to the experimental groups, cells were washed and incubated with DCFH-DA to stain ROS. Following washing, CLSM was used to observe intracellular ROS levels.

**Anti-Inflammatory Evaluation:** Raw 264.7 cells were cultured to 70-80% confluence and stimulated with LPS in different media according to the experimental groups. After incubation, the cells were collected, and total RNA was extracted using a rapid RNA extraction kit. cDNA synthesis was performed using a reverse transcription PCR kit under the following conditions: 25°C for 5 min, 55°C for 15 min, and 85°C for 5 min. Real-time quantitative PCR (RT-qPCR) was performed with SYBR Green Master Mix under these conditions: 95°C for 5 min (initial denaturation), followed by 40 cycles of 95°C for 10 s, 60°C for 20 s, and 72°C for 20 s. The expression levels of inflammation-related genes (IL-1β, IL-6, TNF-α) were determined. Primer sequences are shown in Table 1.

**In Vitro Angiogenesis Evaluation:** Matrigel was melted at 4°C, and pre-cooled pipette tips and plates were used to evenly spread Matrigel onto cell culture plates, avoiding bubbles. The plates were incubated at 37°C to solidify the Matrigel. HUVECs in the exponential growth phase were digested with 0.25% trypsin, resuspended in conditioned media according to the experimental groups, and seeded onto the Matrigel. After 4 hours of incubation at 37°C, capillary-like structure formation was observed under an inverted microscope. ImageJ software was used to quantify the number of nodes, vessel branches, and total tube length.

For gene expression analysis, HUVECs were seeded and incubated with different conditioned media. After incubation, total RNA was extracted, and RT-qPCR was performed to determine the mRNA expression of angiogenesis-related genes (VEGF-A, ICAM-1, VCAM-1). Primer sequences are listed in Table 2.

***In Vivo* Infectious Soft Tissue Repair:** All animal experiments were approved and supervised by the Animal Ethics Committee of Sun Yat-sen University (SYSU-IACUC-2024-B1635, SYSU-IACUC-2024-B1583, SYSU-IACUC-2024-B1634).

Male Sprague-Dawley rats (8-10 weeks, 250-300 g) were randomly divided into four groups: Blank Control, Positive Control, FbHg/AgP, and FbHg/AgP@RFb-Exo. After intraperitoneal anesthesia with sodium pentobarbital, rats were shaved and disinfected. A 1 cm diameter skin defect was created using a surgical punch, and *Staphylococcus aureus* suspension (1 × 10⁸ CFU/mL) was applied to the wound, followed by implantation of the respective materials. The commercially available bio-adhesive (3M Vetbond Tissue Adhesive) was employed for hydrogel fixation.

The next day, wound exudates were collected, diluted with PBS, and cultured on agar plates to count bacterial colonies, confirming successful infection model establishment. A full-thickness mucosal wound was created on the rat palate using a biopsy punch, and treatments were applied according to the groups. Wound healing progress was periodically observed, photographed, and analyzed using ImageJ to calculate the wound closure rate using the formula: Wound closure rate (%) = [(A₀ - Aₜ) / A₀] × 100%. Where: A₀ is the initial wound area. Aₜ is the wound area at time t.

**Histological Analysis:** At designated time points post-surgery, tissues were collected and fixed in paraformaldehyde, dehydrated in graded ethanol, embedded in paraffin, and sectioned. Sections underwent deparaffinization, hematoxylin-eosin staining, and Masson’s trichrome staining. Immunohistochemical staining for IL-1β, TNF-α, and CD31 was performed following antigen retrieval, blocking, and primary/secondary antibody incubation.

***In Vivo* Infectious Hard Tissue Repair:** Male SD rats were anesthetized with sodium pentobarbital. A cylindrical bone defect (4 mm × 4 mm) was created in the medial condyle of the femur using a drill and inoculated with *Staphylococcus aureus* suspension before implanting materials. At 4 weeks post-surgery, samples were collected for micro-CT scanning and histological analysis following decalcification and sectioning procedures as described.

**Statistical Analysis:** Data are presented as mean ± standard deviation (SD). One-way ANOVA was used for multiple group comparisons, and independent sample t-tests were used for two-group comparisons. Statistical significance was set as ns, P>0.05, * P < 0.05, ** P < 0.01, *** P < 0.001. Analyses were performed using GraphPad Prism 9.0 (GraphPad Software, CA).

**Reference**

[1] W. Li, L. Jiang, S. Wu, S. Yang, L. Ren, B. Cheng, J. Xia, *Small* **2022**, 18, 2107544.

[2] J. Du, M. D. Cao, S. L. Feng, F. Su, X. J. Sang, L. C. Zhang, W. S. You, M. Yang, Z. M. Zhu, *Chem–Eur Jl* **2017**, 23, 14614-14622.

[3] K.-C. Kim, M. T. Pope, G. J. Gama, M. H. Dickman, *J Am Chem Soc* **1999**, 121, 11164-11170.


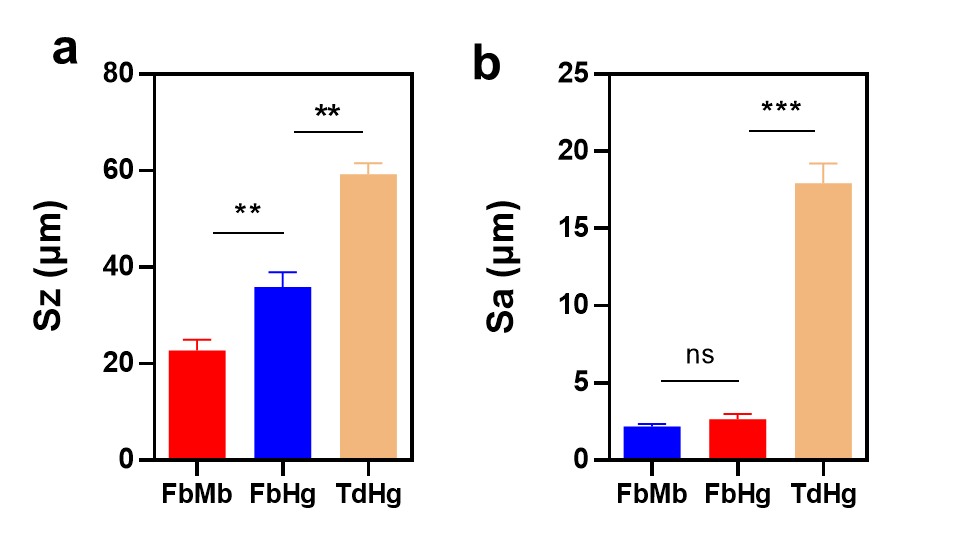


**Figure S1**. (a) Arithmetic mean height deviation (Sz) and (b) maximum height (Sa) of the microprofiles of different samples.


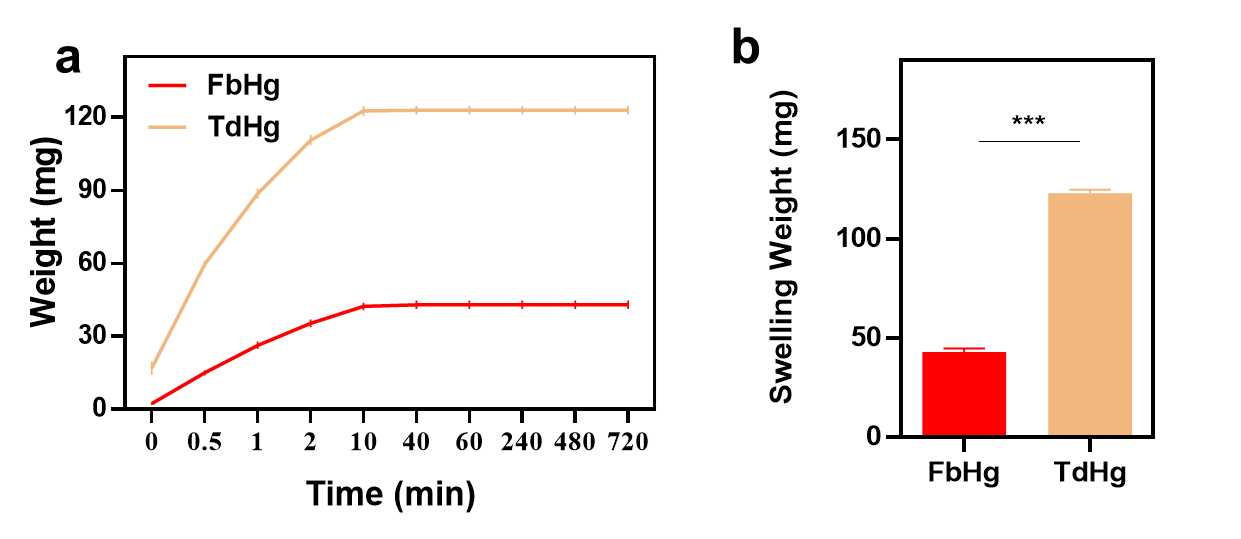


**Figure S2**. (a) Swelling process and (b) equilibrium swelling mass of two hydrogels.

**Figure S3**. Frequency sweep curves of FbMb's rheological performance.

**Figure S4**. Frequency sweep curves of FbHg's rheological performance.

**Figure S5**. Frequency sweep curves of TdHg's rheological performance.

**Figure S6.** Elongation at break for different samples.


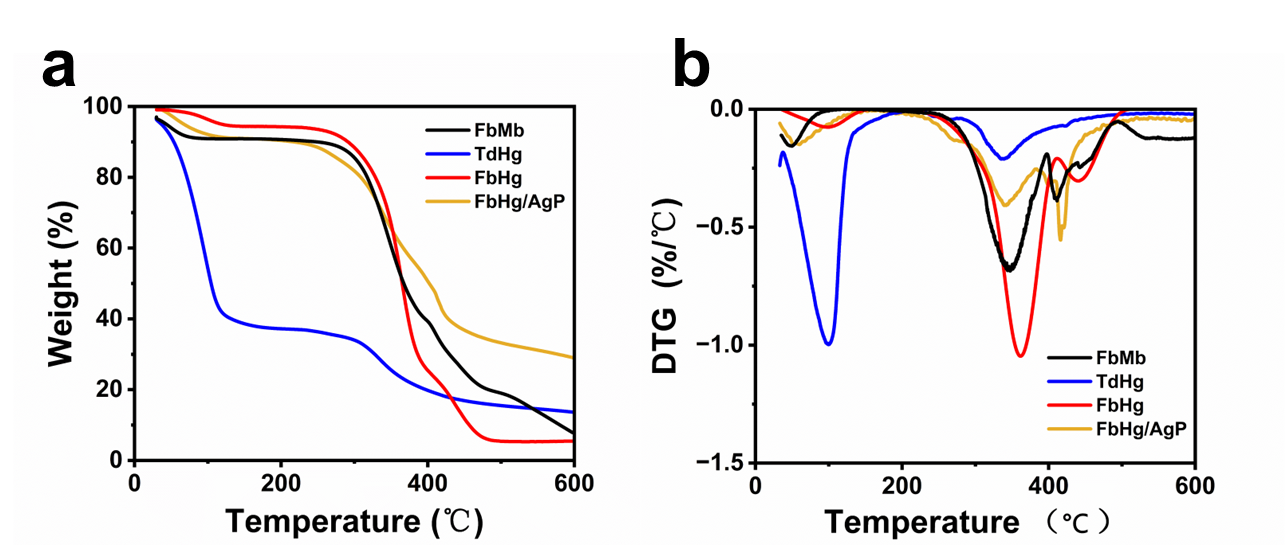


**Figure S7.** Thermogravimetric analysis of hydrogels: (a) TG and (b) DTG curves.


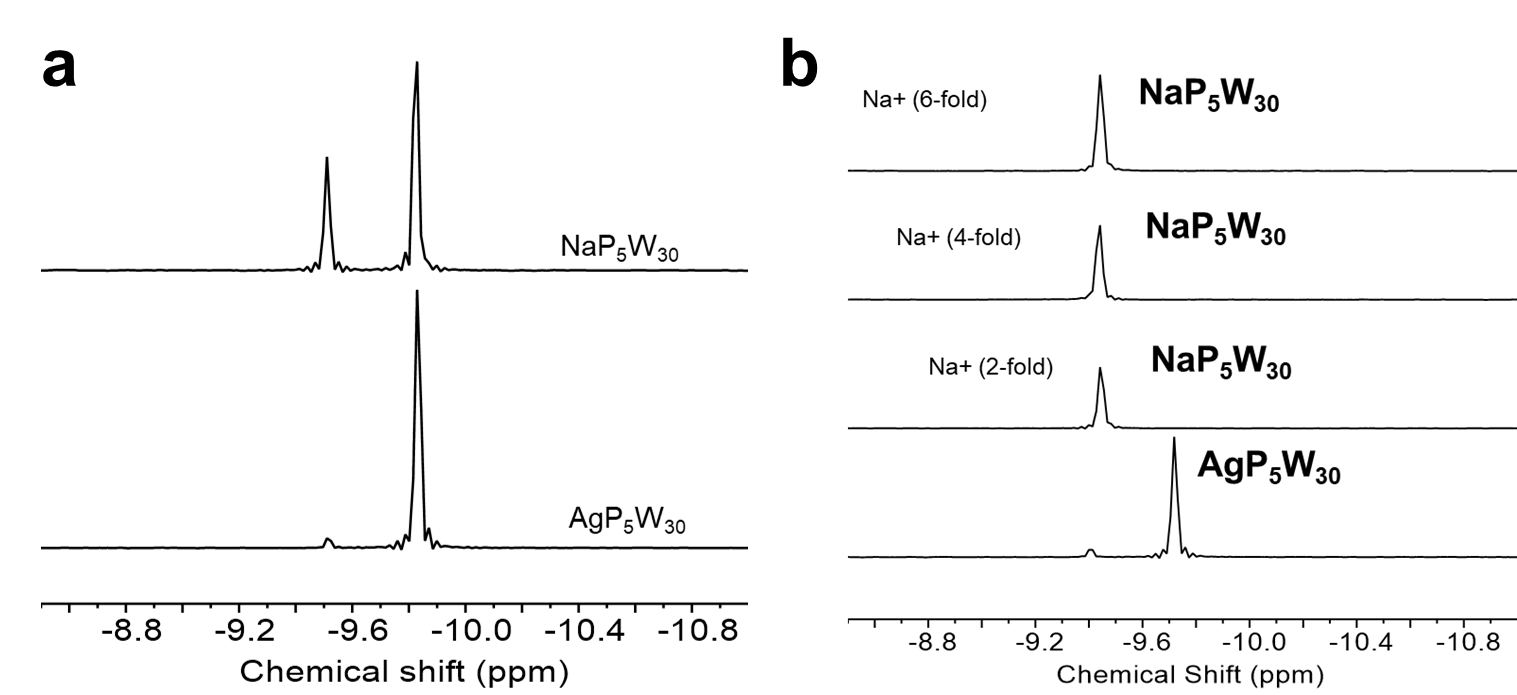


**Figure S8.** ³¹P NMR spectra of (a) AgP_5_W_30_ and NaP_5_W_30,_ (b) The Ag^+^ release behavior regulated by different concentrations of Na^+^.


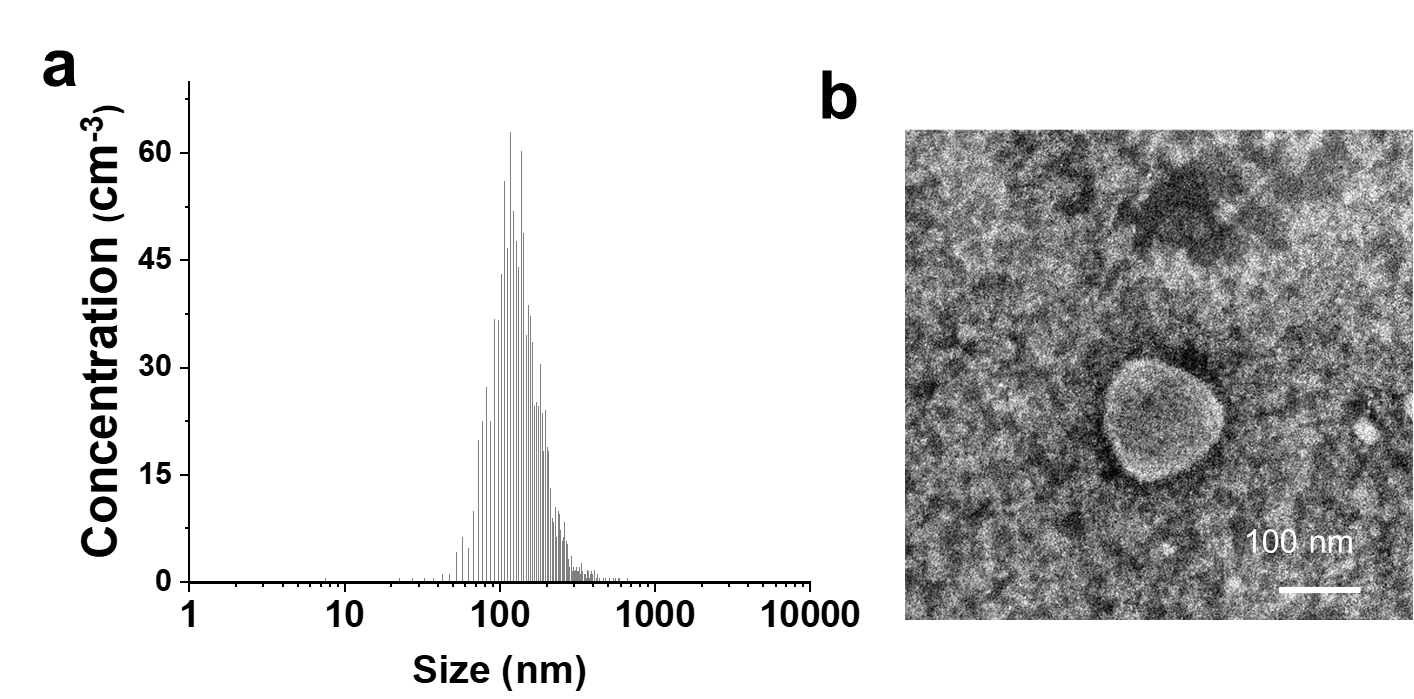


**Figure S9.** (a) Size distribution and (b) morphology of BMSC-derived exosomes.


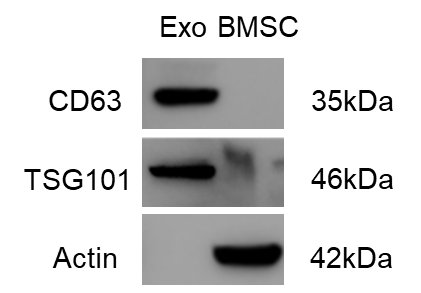


**Figure S10.** Protein markers of BMSC-derived exosomes.


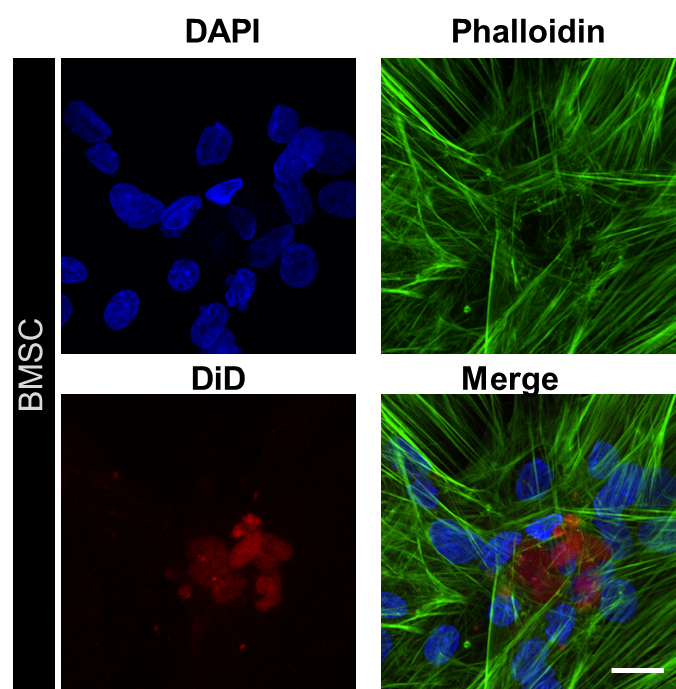


**Figure S11**. Confocal microscopy images of BMSC exosome uptake by cells. Scale bar = 100 μm.


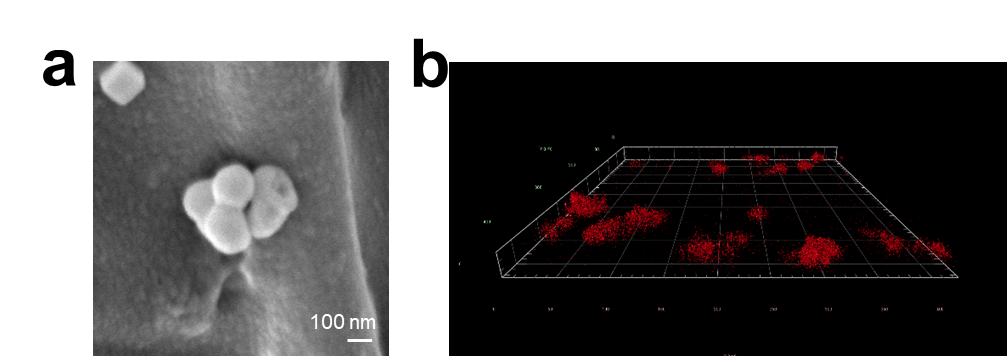


**Figure S12.** (a) Micromorphology and (b) distribution of BMSC exosomes within hydrogels.

**Figure S13**. *In vitro* release profile of hydrogel-loaded components.


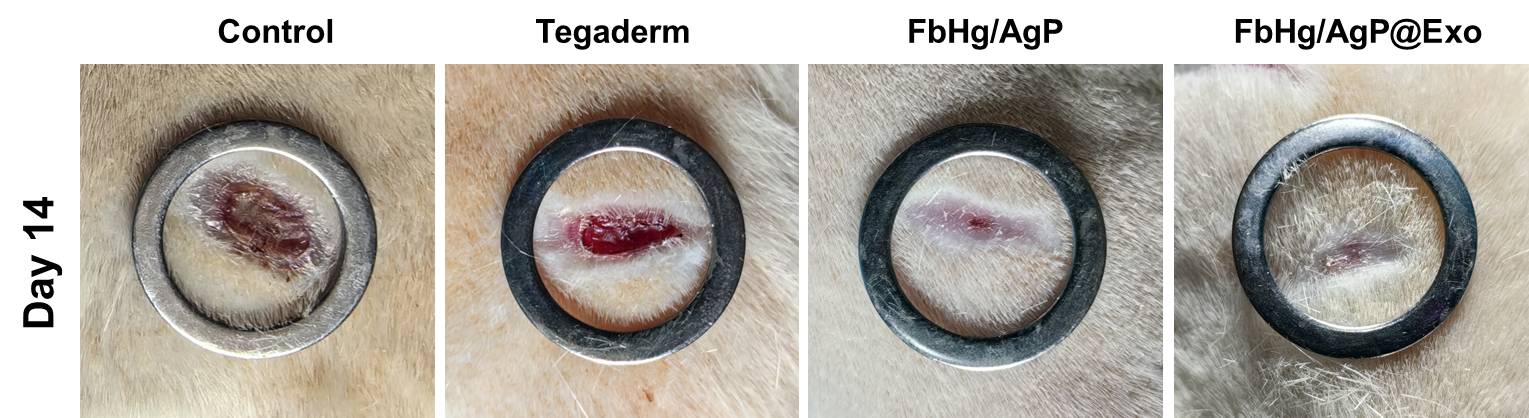


**Figure S14.** Macroscopic images of infection sites at day 14 treated with different hydrogels.

**Figure S15.** Semi-quantitative analysis of Masson's staining in soft tissues.

**Figure S16.** Semi-quantitative analysis of CD31 immunohistochemistry in soft tissues.

**Figure S17.** Semi-quantitative analysis of inflammatory cytokine immunohistochemistry in soft tissues.


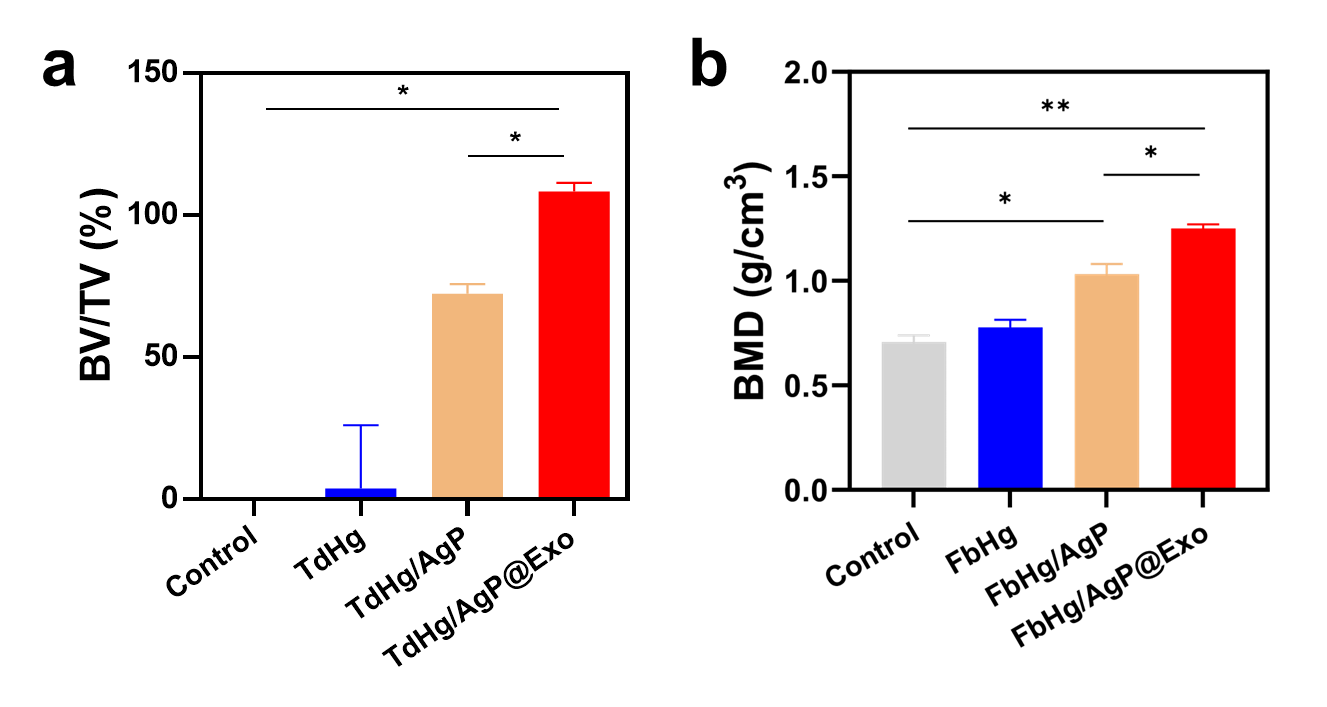


**Figure S18.** Semi-quantitative analysis of (a) Bone volume fraction (BV/TV) and (b) bone mineral density (BMD) from Micro-CT reconstructions.

**Figure S19.** Semi-quantitative analysis of inflammatory cytokine immunohistochemistry in hard tissues.

**Table1.** Main primer sequence

| Gene | Primer sequence (5'-3') | |
| --- | --- | --- |
| mouse-β-actin | Forward | CATCCGTAAAGACCTCTATGCCAAC |
|  | Reverse | ATGGAGCCACCGATCCACA |
| IL-1β | Forward | ACCTGTGTCTTTCCCGTGG |
|  | Reverse | ATGGAGCCACCGATCCACA |
| IL-6 | Forward | AGTTGTGCAATGGCAATTCTGA |
|  | Reverse | AGGACTCTGGCTTTGTCTTTCT |
| TNF-α | Forward | CTGAACTTCGGGGTGATCGG |
|  | Reverse | GGCTTGTCACTCGAATTTTGAGA |

**Table2.** Main primer sequence

| Gene | Primer sequence (5'-3') | |
| --- | --- | --- |
| human-β-actin | Forward | CTCGCCTTTGCCGATCC |
|  | Reverse | TCTCCATGTCGTCCCAGTTG |
| VEGF-A | Forward | AGGGCAGAATCATCACGAAGT |
|  | Reverse | AGGGTCTCGATTGGATGGCA |
| ICAM-1 | Forward | TTGAACCCCACAGTCACCTAT |
|  | Reverse | CCTCTGGCTTCGTCAGAATCA |
| VCAM-1 | Forward | GCTGCTCAGATTGGAGACTCA |
|  | Reverse | CGCTCAGAGGGCTGTCTATC |
